# Safety of poly-L-lactic acid (New-Fill^®^) in the treatment of facial lipoatrophy: a large observational study among HIV-positive patients

**DOI:** 10.1186/1471-2334-14-474

**Published:** 2014-09-01

**Authors:** Martin Duracinsky, Pascale Leclercq, Susan Herrmann, Marie-Odile Christen, Marc Dolivo, Cécile Goujard, Olivier Chassany

**Affiliations:** Bicêtre Hospital, Internal Medicine and Clinical Immunology, University Paris Diderot, Sorbonne Paris Cité, EA REMES, Patient Reported Outcomes Unit, Paris, & AP-HP, Le Kremlin-Bicêtre Paris, France; Grenoble University hospital, Michallon hospital, Information and Treatment Centres for Human Immunodeficiency, BP 217, 38043, CEDEX 09 Grenoble, France; Institute for Immunology and Infectious Diseases, Murdoch University, Perth, Western Australia Australia; Institut Behavior, 27 avenue Marceau, 75116 Paris, France; Association des Œuvres Sociales du Ministère des Affaires Etrangères, 57 Boulevard des Invalides, 75007 Paris, France; Bicêtre Hospital, Internal Medicine and Clinical Immunology, & University Paris Sud, Le Kremlin-Bicêtre Paris, France; Department of Clinical Research, University Paris Diderot, Sorbonne Paris Cité, EA REMES, Patient Reported Outcomes Unit & AP-HP, Saint-Louis Hospital, Paris, France

**Keywords:** HIV, Lipoatrophy, Poly-L-lactic acid, PLLA, Safety

## Abstract

**Background:**

Facial lipoatrophy is a frequently reported condition associated with use of antiretroviral (ARV) drugs. Poly-L-lactic acid (PLLA) acid has been used to correct facial lipoatrophy in people with HIV since 2004 both in Europe and the United States. The objective of this study was to establish, in real life conditions and in a large sample, the safety of PLLA (New Fill^®^, Valeant US, Sinclair Pharma Paris, France) to correct facial lipoatrophy among HIV-positive patients.

**Methods:**

A longitudinal study was conducted between 2005 and 2008 in France. Data from 4,112 treatment courses (n = 4,112 patients) and 15,665 injections sessions (1 to 5 injection sessions per treatment course) were gathered by 200 physicians trained in the use of PLLA.

**Results:**

The average age of patients (88.3% males) treated for lipoatrophy was 47.1 ± 8.1 years (Mean ± SD); 91.2% of patients had been receiving ARV treatment for 10.9 (±4.2) years; CD4 T-cell count was 535 ± 266 cells/mm3. The duration of facial lipoatrophy was 5 ± 2.8 years and the severity was such that 47.3% of patients required five injection sessions of PLLA and 81.9% of the sessions required two vials of the preparation. The final visit, scheduled two months after the last injection session, was attended by 66.0% of patients (n = 2,713). 48 treatment courses (2.8%) were discontinued due to adverse events (AEs). The overall incidence of AEs per course was 18.8%. Immediate AEs, bleeding (3.4%), bruising (2.3%), pain (2.0%), redness at injection site (1.6%), and swelling of the face (0.7%), occurred in 15.4% of courses and 7.0% of sessions (usually during the first session). Non-immediate AEs, mainly nodules (5.7%), inflammation (0.7%), granuloma (0.3%), discolouration (0.2%), and skin hypertrophy (0.1%), occurred in 6.7% of courses. Non-immediate AEs occurred within a time ranging from 21 days (inflammation) to 101 days (granuloma) and all but three of the 13 cases of granuloma resolved. Product efficacy was rated satisfactory by 95% of the patients and physicians.

**Conclusions:**

This study demonstrated, in real-life conditions and on a large sample, that PLLA injections were feasible, efficient, and safe when performed by trained physicians.

**Electronic supplementary material:**

The online version of this article (doi:10.1186/1471-2334-14-474) contains supplementary material, which is available to authorized users.

## Background

Lipodystrophy syndrome is a cluster of long term side effects associated with antiretroviral (ARV) therapy used for human immunodeficiency virus (HIV) infection [1]. The syndrome is associated with lipohypertrophy, characterised by enlargement of the dorsocervical fat pad, thickening of the neck, breast enlargement, abdominal visceral fat accumulation, and lipomata, and/or lipoatrophy, manifested as fat loss from the face, arms, legs, buttocks, and the trunk. Morphological changes are commonly accompanied by metabolic disorders (hyperlipidaemia, insulin resistance and hyperglycaemia). Approximately half of all patients treated for HIV in France are affected with lipoatrophy [2] and choice of treatment for these individuals is largely complicated by this condition [3].

The prevalence of facial lipoatrophy is high. In a recent cross-sectional study in France 54% of patients treated for a median of 10 years presented facial lipoatrophy, and 28% of patients had been receiving treatment for less than 5 years [2]. Facial lipoatrophy has a negative impact on Health Related Quality of Life (HRQoL) [4, 5] and stigma related to altered facial features can lead to suboptimal adherence to ARV treatment [6]. For these reasons, correction of facial lipoatrophy is an integral part of the management of HIV positive patients who present with this problem [7].

To date there is no curative medical treatment for facial lipoatrophy; therapeutic options are limited to cosmetic surgery using autologous adipose tissue, or injections of fillers that may be permanent or absorbable to plump sunken cheeks [8]. New Fill^®^, poly-L-lactic acid (PLLA, Valeant US, Sinclair Pharma, Paris, France), is a biocompatible, resorbable, sterile, non-pyrogenic product introduced to the European market and used in cosmetic surgical procedures in France since 1999, and to correct facial lipoatrophy in people with HIV in Europe and the United States since 2004 where it is administered via a deep intradermal injection. Currently New Fill^®^ is the only product reimbursed in France in this indication [9].

Despite this apparently widespread usage, to the best of our knowledge, no study has evaluated the safety of this treatment in real-life conditions in a large number of people with HIV affected by facial lipoatrophy. Therefore, the aim of this study was to establish the safety, efficacy, and conditions for use of PLLA in that population. The ancillary study of this cohort evaluating Health Related Quality of Life (HRQoL) in 230 of the 4,112 patients included in the current study, was published elsewhere [10]: and the data showed that 64.4% of the patients reported HRQoL improvement greater than 10% 2 months after the procedure, and 58.8% at 12–18 months with 95% of both patients and investigators reporting satisfaction with the PLLA treatment.

## Methods

### Study design and patients

This was an observational, longitudinal, multicentre, open label study conducted in France from 25 February 2005 to 28 February 2008. While the primary objective was to describe the safety of Poly-L-lactic acid for treatment of facial lipoatrophy in the context of HIV, the efficacy of the treatment, the description of its use in real life and the adequacy of prescription compared with the recommendations of the French Commission for the Evaluation of Products and Services (EPSA) [11] were secondary objectives.

The study was conducted in accordance with EPSA requirements. All French physicians trained in the use of PLLA were invited to participate; those agreeing were provided with systematic monitoring notebooks to document information related to the procedure on all patients treated with PLLA. The prerequisite that any physician wishing to prescribe PLLA in France should undergo training facilitated the ascertainment of patients in this study since the researchers were then able to contact all physicians enrolled as PLLA prescribers, seek their participation in the study and utilize the data gathered systematically in usual care through monitoring notebooks.

Verbal informed consent was obtained for all patients by investigators. The study was approved by the Paris Ile de France 4 Ethics Committee, Paris, France. It was conducted in compliance with the Helsinki Declaration [12], with the French Data Processing and Liberties Law no 78.17 of 06.01.1978, received a favourable opinion from the CCTIRS (French Consultative Committee on Data Processing in Research in the field of Health), and was authorised by CNIL (French National Commission on Data Processing and Liberties).

### Data collection

Follow-up visits were planned at the beginning of each course before the first injection of PLLA, then at each injection session, and finally, two months after the end of treatment, representing a treatment course corresponding to the entire process by PLLA for a given patient.

At baseline, the patient demographics, date of HIV diagnosis, the duration of facial lipoatrophy, current ARV therapy, CD4+ T-lymphocyte count, HIV viral load, and the history of previous treatments by the PLLA before this study were collected by physicians in the systematic monitoring notebook.

At each injection session, the physician recorded the conditions of PLLA reconstitution and the number of vials used. Adverse side-effects, which may have been related to the injections and including those that occurred subsequently to the previous session, were also documented. Two months after the last injection, both the physician and the patient gave a subjective assessment of the effectiveness of the treatment on a four-point scale (very satisfied to very dissatisfied) and made a decision whether they would repeat the treatment or not within 12 to 18 months. If the treatment was stopped before the recommended five sessions of injections the reasons, including intolerability, were collected.

The collection of data was consistent for all patients and patients undergoing more than one course of treatment could be included in the study.

### Treatment

A complete treatment course included one to five sessions of injections, each one month apart. At each treatment session the physician injected one or two vials of PLLA, depending on the severity of lipoatrophy, into the dermis. Consistent with the product instructions current at the initiation of the study, the product was to be reconstituted at least 2 hours prior to administration with a recovery window of 2 to 72 hours, using 3 to 5 ml of sterile water for injection by 150 mg PLLA vial.

### Data analysis and statistics

Analyses focused on the data set of patients who received at least one injection session. The mean, standard deviation (SD), median, and first and third quartiles (Q1-Q3) were used to present continuous variables; categorical data were summarized by the number (N) and the percentage of each response category.

Adverse events (AEs) were classified into 2 categories: (1) immediate AEs, contemporary to injections or (2) non-immediate AEs, of later onset, resulting from the effect of several successive injections sessions. Immediate AEs reported in the summary of product characteristics and listed in the monitoring notebook were: bleeding, pain, redness at injection site, bruising, and swelling of the face; non-immediate AEs reported in the summary of product characteristics and listed the notebook were: nodules (i.e., nodules, papules, induration), inflammation, discolouration, granuloma, allergic reaction, skin hypertrophy, and skin atrophy. Non-listed AEs that were reported during the study were classified as “Other AE.” The impact of immediate AEs is presented by injection session and by treatment course (as treatment courses could vary in the number of injections) and the impact of non-immediate AEs by course, since non-immediate AEs could not be attributed with certainty to a particular session.

The impact of the length of the training of physicians and the injections rank number (1^st^, 2^nd^…) on immediate AEs was explored using a mixed model [13] with the “doctor” and “course of treatment” as random effects and “the injection rank number” and “length of training” (<1 year, 1–3 years, > 3 years) as fixed effects. Similarly, the number of non-immediate AEs was explored using a mixed model with the “doctor” as a random effect and the “length of training” as a fixed effect. Similar models have been used to study the impact of the volume of water used for reconstitution (<3, 3–5, > 5 ml) on the number of AEs. Statistical analyses were performed using SAS software release 8.02 (SA Institute, Cary, NC) and AdClin version 3.0 (AdClin, Paris, France).

## Results

Of the 386 physicians trained in the use of PLLA, 272 actually treated patients with PLLA, and 200 returned at least one fully completed systematic monitoring notebook. Physicians were dermatologists (46.5%), plastic surgeons (42.4%), infectious disease specialists (4.5%), or general practitioners (6.6%). The majority (59.7%) had been trained in the use of PLLA for less than a year (mean ± SD: 1.7 ± 2.5 years, median: 0.5 years, Q1-Q3: 0.1 to 2.2 years). The characteristics of the 200 physicians participating in the study were comparable to the whole sample of trained physicians in France.

**Figure 1:**
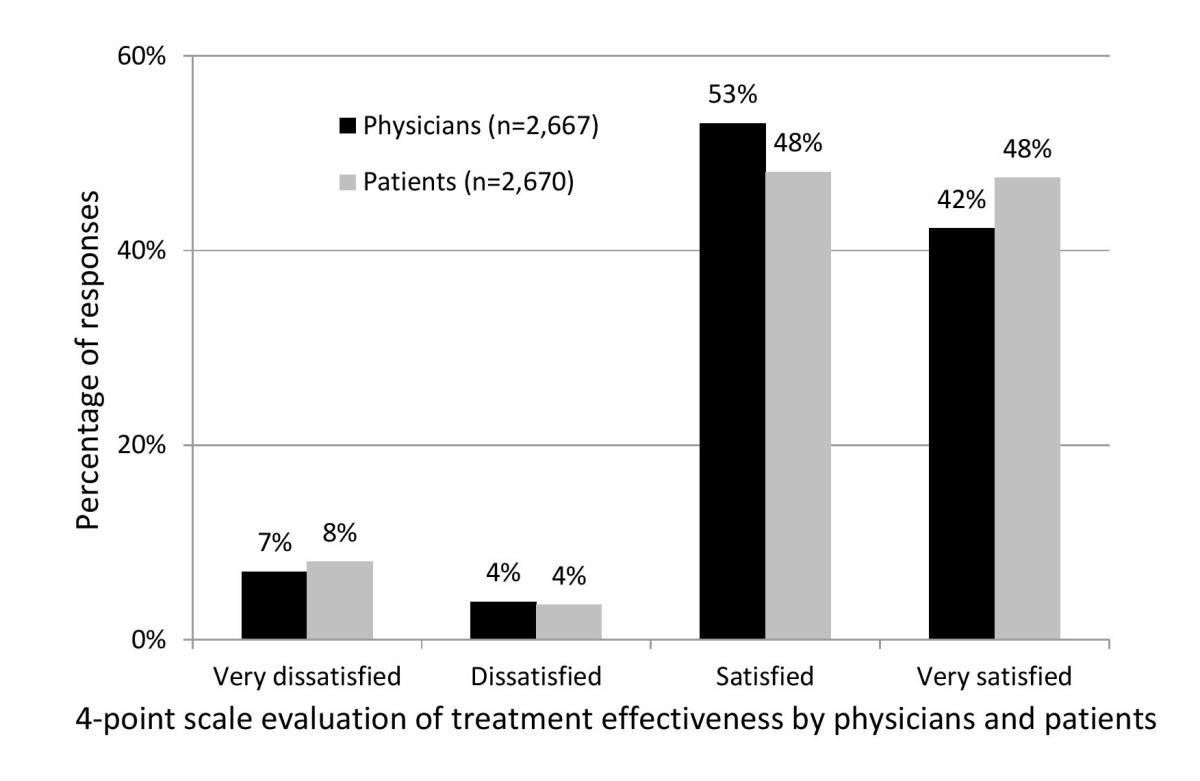
**Subjective evaluation of treatment effectiveness by patients and physicians** **(percentage).**

Data from 4,112 treatment courses (i.e., 4,112 HIV-positive patients) and 15,665 injection sessions were analysed. The vast majority (88.3%) of patients were men. Their median age was 45.8 years (mean ± SD: 47.1 ± 8.1 years). At baseline, the mean time since first diagnosis of facial lipoatrophy was 5 years (5 ± 2.8 years), that of HIV seropositivity was 15.3 (15.0 ± 4.7 years), and duration of ARV drug treatment, 10.9 years (10.9 ± 4.2 years). Notably, 8.8% of patients had no history of ARV therapy when they initiated treatment with PLLA. For patients treated with ARV drugs, current regimens included 34% who were receiving a combination of two nucleoside reverse transcriptase inhibitors (NRTIs) and one protease inhibitor boosted with ritonavir; 21% who were receiving two NRTIs and one non-nucleoside reverse transcriptase inhibitors (NNRTI), and 6.7% were taking three NRTIs. Patients included in the study had 85 different ARV regimens. In 70.6% of patients, the viral load was below 400 copies/ml; mean CD4 t-cell count was 535 ± 266 cells/mm^3^. At the commencement of the study 39.7% of patients had a prior history of corrective treatment with PLLA.The product was used in line with recommended conditions. There was a median time between reconstitution and administration of 24 hours (2–72 hours 94.8% of cases) and a median volume recovery of 5 ml (3–5 ml in 80.2%). Two bottles of product were used in 81.9% of injections sessions. The median time between two successive sessions ranged from 35 days between sessions 1 and 2 to 42 days between sessions 4 and 5. The final visit, scheduled two months after the last injection session, took place in 66.0% of cases (2,713 patients) and at a median of 2.3 months after the last injection session (Q1-Q3: 2–3.7 months). The subjective evaluation of the effectiveness of the treatment on the four-point scale during this visit was very satisfactory, physicians judging the result “satisfied” or “very satisfied” in 95.4% of the cases, and patients in 95.6% (Figure 1).

For 47.3% of patients, five sessions of injections were attended (3.8 sessions on average per treatment course), representing one treatment with a total of 15,665 injections sessions. For 79% of 2,168 patients who had less than five sessions, reasons for discontinuation were indicated (n = 1713) and more than one reason for stopping was possible for a single patient. In 54.8% of cases, the result was considered sufficiently satisfactory to stop the course of injections. In the 27.8% of the cases with reasons for discontinuation documented, the treatment was stopped because the patients did not return for follow-up. In only 48 patients (2.8%), was treatment discontinued because of adverse events and these were noted to be: 25 cases of nodule/induration, eight cases of pain, and seven cases of granuloma. During the study one patient suffered from a myocardial infarction and another died. However, the pharmacovigilance investigation subsequently ruled out any association with the PLLA treatment. One or more AEs were reported in 18.8% of the treatment courses; immediate AEs (15.4%) occurred more frequently than non-immediate AEs (6.7%).

From all treatment courses 66% of the records were available at the visit 2 months (Q1-Q3: 2–3.7 months) after the last injection session. The percentage of injection sessions associated with at least one AE was greater in the group with an evaluation available at 2 months after the last injection session (7.7%, n = 11,358 injections) than in the group lost to follow up (5.3%, n = 4,307).

A total of 1,577 immediate AEs that may have been related to the injections were reported during 1,104 of the 15,665 documented injection sessions (overall incidence: 7.0%). Immediate AEs were: bleeding (reported in 3.4% of the sessions), bruising (2.3%), pain (2.0%), redness at the injection site (1.6%), and localized oedema of the face (0.7%). Most of these events resolved spontaneously: a decrease was observed in 92.3% of cases for bruising, 92.6% for pain, 89.8% for redness at injection site, and 96% for swelling of the face (Table 1). An analysis of immediate AEs, performed on 2,808 systematic monitoring notebooks for which all required data were available, showed that the number of AEs decreased significantly with injections within one course: the overall incidence of immediate AEs declined from 9.9% at the first injection session to 4.6% at the fifth injection session (p < 0.0001). There was no relationship between the number of immediate AEs and length of training of physicians (p = 0.08) nor with the volume of water used for reconstitution (p = 0.03; not significant given the level of significance set at 1% to account for the multiplicity of tests). Immediate AEs were reported for 5.7% of injections sessions (3/53) where the volume recovery was less than 3 ml, 7.4% of the sessions where it was between 3 and 5 ml (894/12,165), and 5.6% if more than 5 ml (165/2946).

**Table 1:** **Immediate AEs by injection session** (**n** = **15**,**565**)

	Incidence^1^per injection session			Regression of AEs^2^
Bleeding	529	(3.4%)	[3.1%; 3.7%]	425/455 (93.4%)
Bruising	365	(2.3%)	[2.1%; 2.6%]	299/324 (92.3%)
Pain	319	(2.0%)	[1.8%; 2.3%]	264/285 (92.6%)
Redness at point of injection	257	(1.6%)	[1.4%; 1.9%]	212/236 (89.8%)
Facial oedema	107	(0.7%)	[0.6%; 0.8%]	97/101 (96.0%)

^1^n (%) [CI 95% exact].

^2^n (% calculated on documented data).

Non-immediate AEs which may have been related to injections (294 in total) were reported during 274 of the 4,112 documented treatment courses (6.7%) and were reported as nodule/induration (in 5.7% of courses), inflammation (27 cases, 0.7%), granuloma (13 cases, 0.3%), discolouration (9 cases, 0.2%), skin hypertrophy (6 cases, 0.1%), allergic reaction (3 cases, 0.1%), and skin atrophy (2 cases, 0.0%). The median time to onset was 21 days for inflammation, 56 days for nodule/induration, almost 100 days for hypertrophy skin, discolouration, and granuloma (Table 2). The regression of signs could be observed in all documented cases with the exception of three cases of granuloma. However, this result must be qualified by the large number of missing data (respectively 172/234, 8/27, 4/13, 7/9, 5/6, and 2/3 for nodule/induration, inflammation, granulomas, discoloration, skin hypertrophy, and allergic reaction) (Table 2).

**Table 2:** **Non**- **immediate AEs by treatment course** (**n** = **4**,**112**)

	Incidence^1^per course			Delay of onset (days)^2^	Regression of AEs^3^
Nodule/induration	234	(5.7%)	[5.0 %; 6.4 %]	55 [29 – 91]	62/62 (100%)
Inflammation	27	(0.7%)	[0.4%; 1.0%]	21 [0 – 57]	19/19 (100%)
Granuloma	13	(0.3%)	[0.2%; 0.5%]	101 [69 – 117]	6/13 (46%)
Decolouration	9	(0.2%)	[0.1 %; 0.4%]	92 [11 – 138]	2/2 (100%)
Skin hypertrophy	6	(0.1%)	[0.1%; 0.3%]	84 [40 – 93]	1/1 (100%)
Allergic reactions	3	(0.1%)	[0.0%; 0.2%]	1 [0 – 94]	1/1 (100%)
Skin atrophy	2	(0.0%)	[0.0%; 0.2%]	66 [13 – 119]^4^	-

^1^n (%) [CI 95% exact].

^2^Median [Q1 – Q3] (calculated on documented data).

^3^n (% calculated on documented data).

^4^Reported values for the 2 cases.

A treatment course had a mean of 3.8 injections.

Neither the “duration of the training of physicians” nor the volume of water used for reconstitution effect had a significant impact on the frequency of occurrence of non-immediate AEs.

Besides the expected and listed AEs, 72 AEs considered as possibly related to PLLA injections were classified as “other” (overall incidence: 0.5%). Discomfort (19 cases, 0.1%), tingling (11 cases, 0.1%), hematoma (5 cases, 0.0%), and folliculitis cheeks (4 cases, 0.0%) were the only events reported more than 3 times during the 15,665 injection sessions. Only one case of cutaneous necrosis met the criterion of gravity (according to French Adverse Event reporting procedures) and was reported a serious AE, but the necrosis resolved.

## Discussion

This 3-year observational study (2005–2008) describes the safety, efficacy, and use of PLLA in France *via* a comprehensive collection of information on a large sample of HIV-positive patients presenting with facial lipoatrophy and treated with PLLA. The patients in this observational study were older and more likely to be men than the patients with long-term HIV disease over the period 2006–2008 followed in the French Hospital Database on HIV [3] and the wide variety of ARV regimens reflects the long exposure to ARV treatment. Virological control (<400 copies/ml) was achieved in 70.6% of patients, suggesting that therapeutic success was lower in this population than in comparison with all French ARV treated patients (88% < 500 copies/ml in 2007). This may be explained by the fact that patients in this sample had been exposed for a longer period to antiretroviral drug toxicity. Treatment failure had led to more frequent switches than was seen in the French Hospital Database. Moreover, the lower limit of detectability of viral load in our study was different than that reported for the French population during the same period.

The vast majority of AEs reported were mild and transient and only one serious AE (skin necrosis) was reported. The overall incidence rate of AEs was only around one fifth of treatment courses, and immediate AEs occurred more frequently than non-immediate AEs which were less than seven percent. The immediate impact of AEs decreased with the number of injections sessions and did not depend significantly, on the duration of physicians’ training to use the product, or the volume of reconstitution. Essentially, immediate AEs (bleeding, bruising, pain, redness and oedema) were related to the injection technique and non-immediate AEs could be related to the product itself but arguably also to the product reconstitution or injection technique. Nodule/induration was rare and other non-immediate AEs (granuloma, inflammation, discolouration, allergic reaction, skin atrophy, or skin hypertrophy) occurred in less than 1% of patients. Reasons for discontinuation were reported for 1713 patients and only 48 courses (2.8%) were discontinued due to the occurrence of AEs, mostly non immediate but injection-related AEs may occur with any injectable filler and generally resolve spontaneously.

Prior to this study, 12 studies on the safety of PLLA [14–25] involving a total of 744 HIV-positive patients treated with PLLA with study numbers ranging from 20 patients [17] to 115 patients [24], have been published and no serious AEs associated with the use of PLLA were reported. However, immediate AEs were more frequently reported in these studies than the one reported here. Carey and colleagues (2007) noted that [15] 76% of the patients complained of pain or discomfort, 64% of localised oedema, and 53% of erythema. In the study by Lafaurie et al. [18] 77% of the patients reported pain and 4% bruising or bleeding at the injection site. Moyle et al. [21] reported that 38% of patients in their study had contusions, 10% inflammation, discomfort and erythema, and 7% oedema. While Cattelan et al. [16] observed that 30% of patients had oedema and 24% bruising at the injection site. Finally Mest and Humble [20] noted that 30% of patients had a haematoma. Several reasons might explain these variations such as differential reporting between physicians (e.g. a transient local swelling after subcutaneous injection of 4 ml is unavoidable), different treatment schedules and injection techniques. In our study, immediate AEs were noted after each session. It is likely that weekly evaluations would have been more precise for assessing non-immediate AE. However, since this was an observational cohort study without modification of usual clinical care, a one month recall period was chosen.

Nodule is the most commonly observed non-immediate AE observed in the cosmetic procedure, but with varying frequency depending on the study. Orlando et al. [24] reported that 45% of the 91 patients followed for 48 weeks had invisible but palpable, subcutaneous micronodules, nearly eight times higher than in our study. Palpable, intraoral, intradermal papules were reported in 8 of 20 patients by Guaraldi et al. [17] and in 9 of 30 patients by Moyle et al. [21]. Three other studies have reported nodules with a frequency of about 10% [15, 18, 22]. These authors also reported cases of papules at the injection site in 12% of patients, as did Mest and Humble [20]. Other studies have reported this type of AE with a lower frequency, if at all [16, 19, 25]. In our large-scale real life study, physicians reported the frequency of nodules at 5.7%.

The usage of PLLA across Europe and the United States for the correction of cutaneous depressions became widespread. The safety profile of PLLA has improved dramatically over recent years with increased experience in product preparation, in selection of injection area and in injection modalities. In particular a decreased risk of adverse reactions with an increased dilution was noted in several clinical trials and in the Injectable Filler Safety Study [26].

Our study has a number of limitations. Of the 272 physicians who treated patients with PLLA and agreed to participate to the study, 72 did not return notebooks but we also had no knowledge of whether a physician started a notebook but did not submit it. In addition, 34% of patients were not evaluated 2 months after the last injection. However, it is most likely that the occurrence of adverse effects would have encouraged the patient to return to the trained doctor rather than to another who was not familiar with the use of the product and did not perform the procedure.

Nevertheless, the strength of this study is the large sample size, over 4,000 people treated with PLLA versus less than 100 for most other published studies and with only a small number of discontinuations for intolerable adverse effects. Furthermore, the safety of this treatment for the correction of lipoatrophy in people with HIV has been demonstrated *in real life* conditions.

## Conclusions

This prospective, observational study examined the safety of poly-L-lactic acid for the correction of facial lipoatrophy in HIV patients. This was a large study involving more than 4,000 treatment courses, and 15,000 injection sessions of PLLA were used to treat patients with severe facial lipoatrophy. One quarter of the patients required five treatment sessions and 82% of sessions required the use of two vials of PLLA. The subjective efficacy of the treatment was reported as “satisfied” or “very satisfied” by most patients and physicians. The product was well tolerated and the incidence of immediate and non-immediate AEs was acceptable. The vast majority of AEs reported were mild and transient and immediate AEs occurred more often during the first injection session than in subsequent sessions. Nodule/induration was rare and granuloma occurred seldomly. Less than 3% of the PLLA treatments were discontinued due to an adverse event, despite the large number of injections carried out, and no new adverse events of PLLA were described. We have demonstrated in real-life conditions that, in the hands of trained physicians, treatment of ARV toxicity-induced facial lipoatrophy with poly-L-lactic acid (New Fill^®^) is feasible, efficient, and safe in clinical practice.

## Authors’ original submitted files for images

Below are the links to the authors’ original submitted files for images. Authors’ original file for figure 1

## Abbreviations

AE: Adverse Event
ARV: Antiretroviral
CNIL: French National Commission on Data Processing and Liberties
CCTIRS: French Consultative Committee on Data Processing in Research in the field of Health
EPSA: French Commission for the Evaluation of Products and Services
HIV: Human Immunodeficiency Virus
HRQoL: Heath Related Quality of Life
NRTI: Nucleoside Reverse Transcriptase Inhibitors
NNRTI: Non-Nucleoside Reverse Transcriptase Inhibitor
PLLA: Poly-L-Lactic Acid
Q: Quartile
SD: Standard Deviation.
