# Supplementary figures and images for: Safety of poly-L-lactic acid (New-Fill®) in the treatment of facial lipoatrophy: a large observational study among HIV-positive patients
Source: BMC Infect Dis. 2014 Sep 1;14:474. doi: 10.1186/1471-2334-14-474 (PMC4160543; doi:10.1186/1471-2334-14-474)

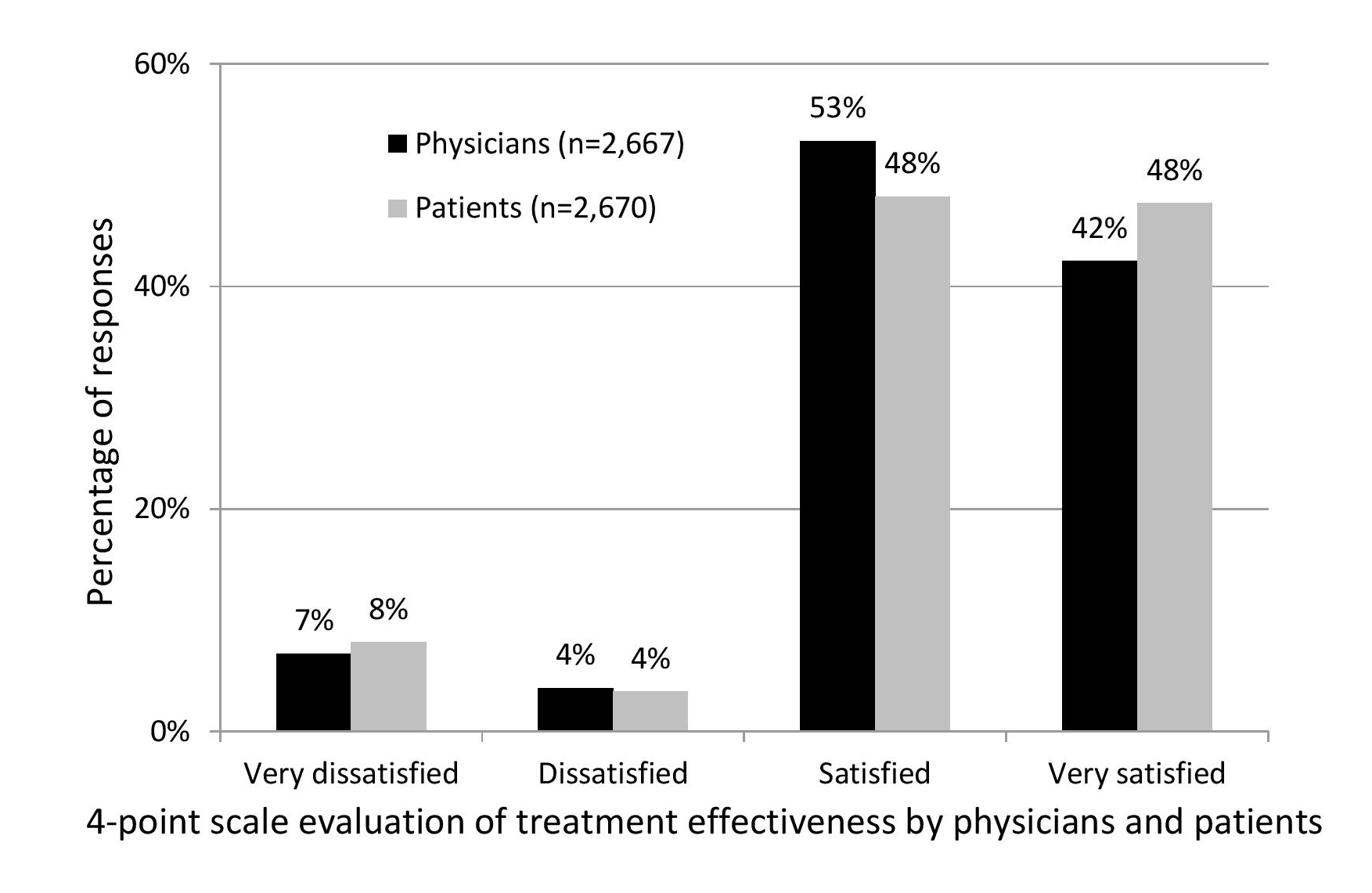

Supplement: Supplementary file 1 — Authors’ original file for figure 1 [file 12879_2014_3780_MOESM1_ESM.png]
